# Multi‐locus genetic basis of stomatal density and size in maize revealed by integrated QTL and GWAS

**DOI:** 10.1111/tpj.71187

**Published:** 2026-11-02

**Authors:** Yongtian Qin, Meng Qiu, Chuyang Wang, Zeyuan Yang, Xurui Ma, Zhaoyi Pan, Lingling Sun, Tingting Wu, Yuqi Huang, Hongqiu Wang, Qingqian Zhou, Jihua Tang, Zhiyuan Fu

**Affiliations:** ^1^ State Key Laboratory of High‐Efficiency Production of Wheat‐Maize Double Cropping/College of Agronomy/The Shennong Laboratory Henan Agricultural University Zhengzhou 450046 China; ^2^ Hebi Academy of Agricultural Sciences Hebi 458030 Henan China; ^3^ Zhoukou Polytechnic Zhoukou 466000 China

**Keywords:** maize, stomatal traits, linkage and GWAS mapping, candidate gene validation, selection

## Abstract

Stomatal patterning is a fundamental aspect of plant photosynthetic performance and water‐use efficiency, yet its genetic basis in C_4_ grasses such as maize remains poorly understood. Here, we integrated linkage analysis across three recombinant inbred line populations and a genome‐wide association study in a diverse maize panel to dissect the genetic architecture of six stomatal traits. This approach identified 33 quantitative trait loci and 51 association loci for 6 stomatal traits and revealed a significant negative genetic correlation between stomatal density (SD) and size, with multiple genomic intervals showing co‐localization of effects on both trait categories. By integrating reported cell‐type‐specific transcriptomic data, we prioritized high‐confidence candidate genes, including conserved stomatal regulators as well as previously uncharacterized candidates involved in processes such as cell wall biosynthesis. Functional analyses of EMS‐induced mutants for candidates *ZmbHLH140* and *ZmCESA6* demonstrated that loss of *ZmbHLH140* reduces SD, whereas mutation of *ZmCESA6* has the opposite effect. In addition, the signature of selection at the *ZmbHLH140* locus is consistent with a role for stomatal regulation during maize domestication. Together, our results establish a comprehensive genetic framework for stomatal development in maize, highlighting both conserved and grass‐specific mechanisms that shape stomatal patterning and offering a foundation for future functional studies of stomatal development in cereals.

## Significance Statement

Stomatal patterning affects photosynthesis and water use, yet its genetic basis in maize has been poorly understood. By combining linkage analysis and genome‐wide association study (GWAS) across diverse populations, we identified 33 quantitative trait loci (QTL) and 51 loci, uncovered density–size trade‐offs, and validated opposing functions of ZmbHLH140 and ZmCESA6 on stomatal density, establishing a framework for understanding stomatal development in cereals.

## INTRODUCTION

Stomata, the microscopic pores on the leaf epidermis, regulate plant gas exchange by balancing photosynthetic CO_2_ uptake with transpirational water loss (Hetherington & Woodward, 2003; Lawson & Blatt, 2014). Given this essential role, stomatal patterning is relevant to crop productivity, water‐use efficiency, and stress resilience (Fanourakis et al., 2015; Faralli et al., 2019). In grasses, stomatal function is supported by a specialized stomatal complex composed of dumbbell‐shaped guard cells flanked by subsidiary cells arranged in discrete files (Chen et al., 2017; Hepworth et al., 2018; Nunes et al., 2020). This unique anatomy is thought to underlie the high rates of gas exchange characteristic of C_4_ species such as maize (Taylor et al., 2010, 2012).

The genetic framework controlling stomatal development has been most extensively characterized in the C_3_ model plant Arabidopsis. In this system, stomatal lineage initiation and differentiation are controlled by a conserved signaling and transcriptional network involving EPIDERMAL PATTERNING FACTOR (EPF) family signaling peptides, the receptor‐like kinase TOO MANY MOUTHS (TMM), ERECTA (ER)‐family receptor kinases, and the basic helix–loop–helix (bHLH) transcription factors SPEECHLESS (SPCH), MUTE, and FAMA, together with their heterodimeric partners INDUCER OF CBF EXPRESSION1 (ICE1) and SCREAM2 (SCRM2), that orchestrate cell fate transitions (Chen, 2023; de Marcos et al., 2016; Lee et al., 2015; Lin et al., 2017; Liu et al., 2009; Nadeau & Sack, 2002; Pillitteri et al., 2007; Shpak et al., 2005; Von Groll et al., 2002; Zoulias et al., 2018). Orthologs of several key regulators (e.g., SPCH, MUTE, FAMA) are functionally conserved in grasses, indicating that fundamental aspects of stomatal lineage control are shared across angiosperms (Apostolakos et al., 2018; Hepworth et al., 2018; Hughes et al., 2023; Liu et al., 2009; McKown & Bergmann, 2020; Raissig et al., 2016, 2017; Shaar‐Moshe & Brady, 2023; Wang et al., 2019; Wu et al., 2019; Yu et al., 2020; Zhou et al., 2025).

Despite this conservation, stomatal development in maize and other grasses displays distinctive features that are not fully explained by the Arabidopsis‐derived framework. The formation of subsidiary cells, the spatial organization of stomata into files, and the integration of actin dynamics and polarized cell divisions rely on grass‐specific regulators, such as PAN1 (LRR‐like protein Pangloss1) and PAN2 (Cartwright et al., 2009), actin‐associated proteins ROP2 and ROP9 (Rho of plants), and the ARP2/3 complex (Actin‐Related Proteins 2/3) (Facette et al., 2015; Humphries et al., 2011), and specialized transporters (guard cell‐ and subsidiary cell‐specific Shaker type K^+^ channels, ZMK, KZMs, ZORK, and ZmSLACs) (Mumm et al., 2011; Wolf et al., 2006) that are absent from Arabidopsis. As a result, the extent to which conserved stomatal regulators interact with maize‐specific pathways to shape stomatal patterning and morphology remains poorly understood.

Stomatal density (SD) and stomatal size (SS) have emerged as particularly attractive targets for crop improvement. However, the functional relevance of these traits is nuanced; a directly relevant quantitative trait loci (QTL) mapping study in maize found that stomatal conductance was genetically correlated with stomatal complex dimensions but not with density (Xie et al., 2021), and live imaging has shown that patterning alone does not determine conductance (Crawford et al., 2025). Despite these complexities, genetic manipulation of stomatal traits has been shown to contribute to drought tolerance and yield in cereals such as rice and wheat (Caine et al., 2019, 2023; Huang et al., 2009; Mohammed et al., 2019; Zhao et al., 2022). However, SD and SS are often inversely correlated, reflecting a fundamental trade‐off that constrains their independent optimization (Caine et al., 2023; Franks & Beerling, 2009). Dissecting the genetic basis of this trade‐off requires approaches that can resolve potentially pleiotropic loci and distinguish shared from trait‐specific control. Natural variation provides a powerful resource for such analyses, yet previous studies of stomatal traits often relied on single mapping populations or lacked functional genomic integration.

Here, we leverage natural variation in maize to construct an integrated genetic framework for stomatal trait variation. By combining linkage mapping across multiple recombinant inbred line (RIL) populations with genome‐wide association studies (GWAS) in a diverse panel and integrating these analyses with cell‐type‐specific single‐nucleus transcriptomic data (Sun et al., 2022), we identify robust QTL and prioritize candidate genes underlying SD, SS, and arrangement. Functional validation of selected candidates further links genetic variation to stomatal development. Together, this work provides new insight into the genetic architecture of stomatal patterning in maize and establishes a foundation for mechanistic studies.

## RESULTS

### Genetic variation of stomatal traits

To characterize the genetic architecture of stomatal traits in maize, we analyzed three RIL populations derived from crosses between elite inbred lines representing distinct genetic backgrounds: BYD (DE3 × By815), BYK (K22 × By815), and SZ (Z58 × SK). These populations, comprising 105–163 lines each, provide independent resources for linkage mapping and enable the identification of both shared and population‐specific QTL (Pan et al., 2016) (Table S1). In parallel, a diverse association panel of 262 maize inbred lines was used for GWAS to increase mapping resolution and refine candidate loci (Yang et al., 2010) (Table S2). Together, this integrated experimental framework combines complementary genetic approaches to robustly dissect natural variation in stomatal traits (Figure 1a).

**Figure 1:**
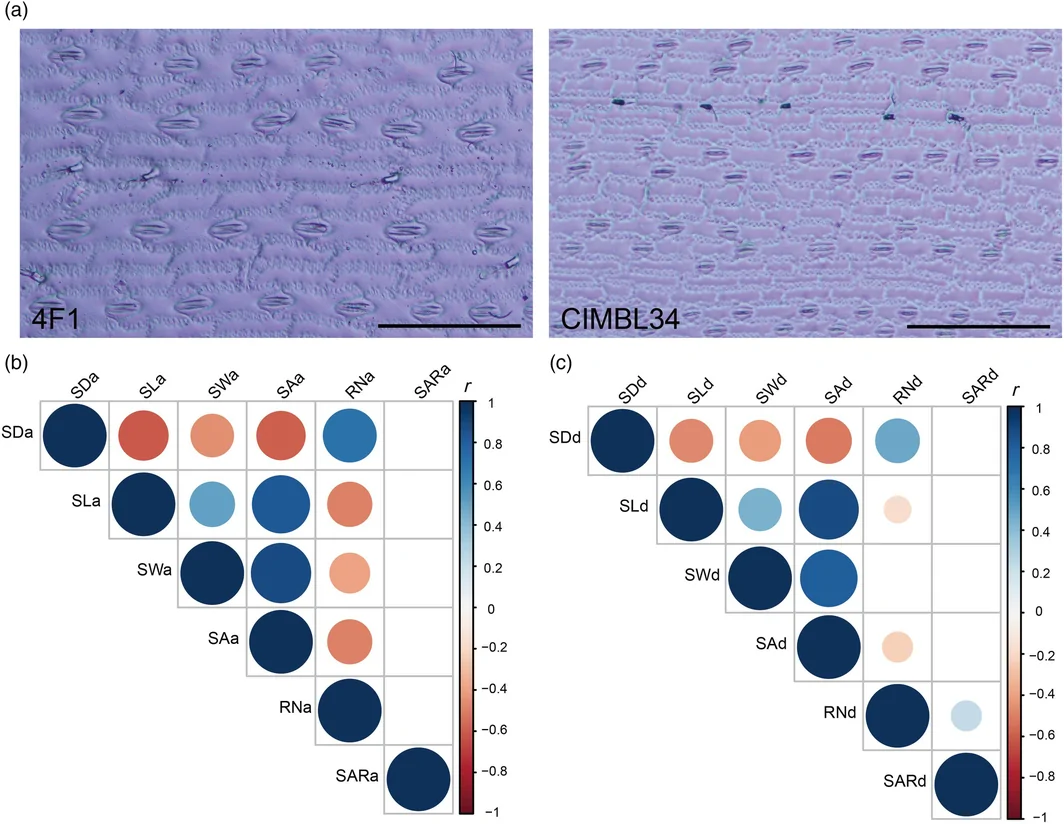
Stomatal phenotypic variation in the mapping populations. (a) Representative stomatal images of the inbred lines. Scale bar = 200 μm. (b, c) Correlation matrices of the six measured stomatal traits in the (b) BYD recombinant inbred line (RIL) population and the (c) association panel. RN, stomatal row number; SA, stomatal area; SAR, stomatal arrangement ratio; SD, stomatal density; SL, stomatal length; SW, stomatal width. The lowercase a and b represent BYD and BYK population, respectively.

We quantified six key stomatal traits—SD, length (SL), width (SW), area (SA), row number (RN), and stomatal arrangement ratio (SAR)—across the three RIL populations. All traits exhibited a broad range of continuous phenotypic variation and followed an approximately normal distribution (Table S3). Among these traits, SD, SA, and RN exhibited high variability with coefficient of variation ranging from 17.18–18.65%, 13.17–15.10%, and 10.02–11.31%, respectively, whereas SL, SW, and SAR showed moderate variation with CVs of 7.36–8.03%, 9.12–9.53%, and 3.15–4.66% across different populations (Table S3). Correlation analysis revealed a clear and consistent pattern across all RIL populations. SD was strongly positively correlated with RN but negatively correlated with the size‐related traits SL, SW, and SA (Figure 1b; Figure S1). In contrast, SAR showed no significant correlation with the other measured traits. These relationships are consistent with an intrinsic trade‐off between SD and SS in maize, suggesting potential coordinated developmental regulation rather than independent trait control.

The association panel displayed similarly extensive phenotypic variation and preserved the same correlation structure observed in the RIL populations (Figure 1c; Table S4). In particular, SD was again positively correlated with RN and negatively correlated with SL, SW, and SA, while SAR remained largely independent. The conservation of these trait relationships across multiple biparental populations and a genetically diverse association panel reinforces the robustness of the observed density–size trade‐off and provides a strong foundation for subsequent genetic mapping analyses.

### Identification of QTL for stomatal traits

Linkage analysis across the three RIL populations identified a total of 43 non‐redundant QTL intervals, corresponding to 55 trait–QTL associations for the 6 stomatal traits analyzed at 2.5 LOD threshold (Table S5). These QTL were distributed across all maize chromosomes but were unevenly represented, with chromosomes 1, 2, and 5 harboring the highest number of loci, whereas chromosomes 7 and 8 each contained a single QTL. Individual QTL explained between 5.08 and 16.87% of the phenotypic variance. Fourteen QTL phenotypic variance explained (PVE) greater than 10% were classified as major‐effect loci and were detected on chromosomes 1, 2, 3, 5, and 6 (Figure 2; Figures S2 and S3).

**Figure 2:**
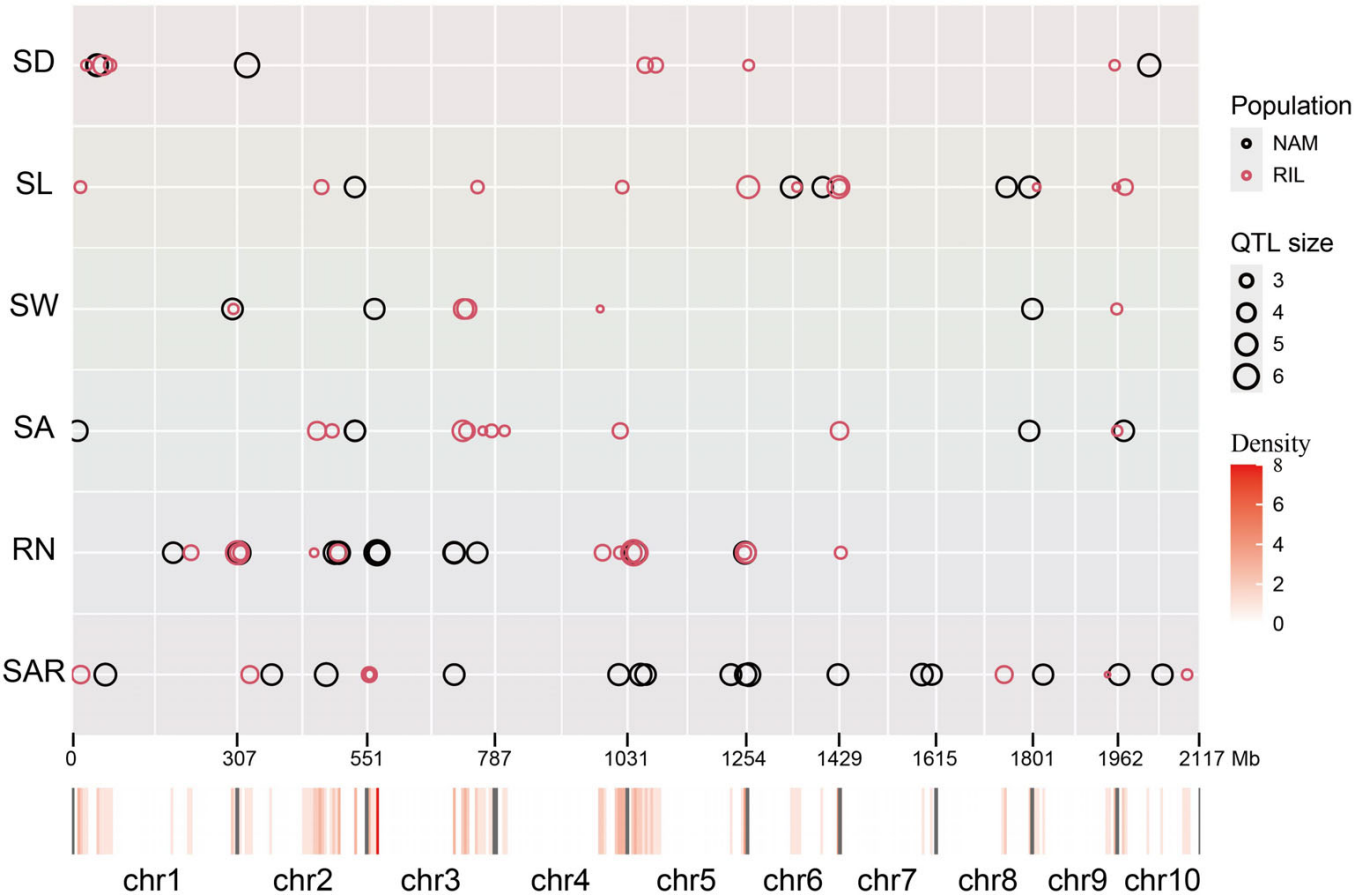
Genome‐wide distribution of quantitative trait loci (QTL) for stomatal traits. The chromosomal locations of QTL identified for stomatal traits through linkage mapping (shown as confidence intervals) and genome‐wide association study (GWAS) (shown as ±30 kb regions flanking the lead single‐nucleotide polymorphism [SNP]) are plotted. Traits: RN, stomatal row number; SA, stomatal area; SAR, stomatal arrangement ratio; SD, stomatal density; SL, stomatal length; SW, stomatal width. The *x*‐axis represents the physical position along the maize chromosomes (Mb). The heatmap below illustrates the density of QTLs across the genome using a 5 Mb sliding window. The color intensity (white to red) corresponds to the number of QTLs within each window. Detailed information for each QTL is provided in Tables S5–S7.

We next focused on robust QTL that satisfied either of the following criteria: they exceeded the permutation‐derived stringent thresholds (LOD 3.1–3.6, 23 QTL intervals), or they were conditionally retained based on independent documentation in at least three published studies of stomatal traits or drought‐stress responses, together with significant enrichment over a permutation‐based null expectation (*P* < 0.05; Figure S4; 10 QTL intervals). These criteria collectively yielded a final set of 33 QTL intervals (Table S5). Several QTL intervals were detected repeatedly across independent RIL populations, indicating genetic robustness. Three genomic intervals were identified in at least two populations, including intervals on chromosomes 1 (*qSL1‐1a*/*qSAR1‐1b*), 2 (*qRN2‐1b*/*qSA2‐1a* and *qRN2‐1b*/*qSL2‐1a*), and 4 (*qRN4‐1b*/*qSA4‐1b*/*qSL4‐1c*). In addition, four intervals in individual populations were associated with multiple SS traits, such as *qSA3‐1c*/*qSW3‐1c*, *qSA3‐2c*/*qSW3‐2c*, and *qSL6‐2a/qSA6‐1a*, suggesting the presence of loci with potential pleiotropic effects influencing coordinated aspects of stomatal morphology (Figure 2; Table S5; Figures S2 and S3).

GWAS in the diverse association panel identified 128 significant non‐redundant single‐nucleotide polymorphisms (SNPs), which defined 51 non‐redundant association loci. Notably, 48 of these loci overlapped with QTL that had been independently documented in at least one prior publication related to stomatal traits or drought‐stress responses (Figure 2; Table S6; Figures S5– S7). Most association loci were trait‐specific, with only a single SNP detected for both SL and SA (chr2.s_214800421). Notably, comparison of linkage‐ and association‐based results revealed six QTL intervals that were independently supported by both approaches (Table S6; Figure 3). These overlapping loci were located on chromosome 5 (*qSD5‐1b/qSD5‐2b*) (Figure 3a,b), chromosome 5 (*qRN5‐1a/qRN5‐2a*) (Figure 3c–f), chromosome 2 (*qRN2‐2b*), chromosome 3 (*qSL3‐1a*), chromosome 4 (*qRN4‐1b*/*qSA4‐1b/qSL4‐1c*), and chromosome 6 (*qSL6‐1a*) (Table S6). Together, the linkage and association analyses reveal a complex but structured genetic architecture underlying stomatal traits in maize. The presence of major‐effect loci, repeated detection across populations, and co‐localization between mapping approaches highlights a subset of robust QTL that are likely to play central roles in controlling natural variation in SD, SS, and arrangement. These high‐confidence loci provide the basis for candidate gene prioritization.

**Figure 3:**
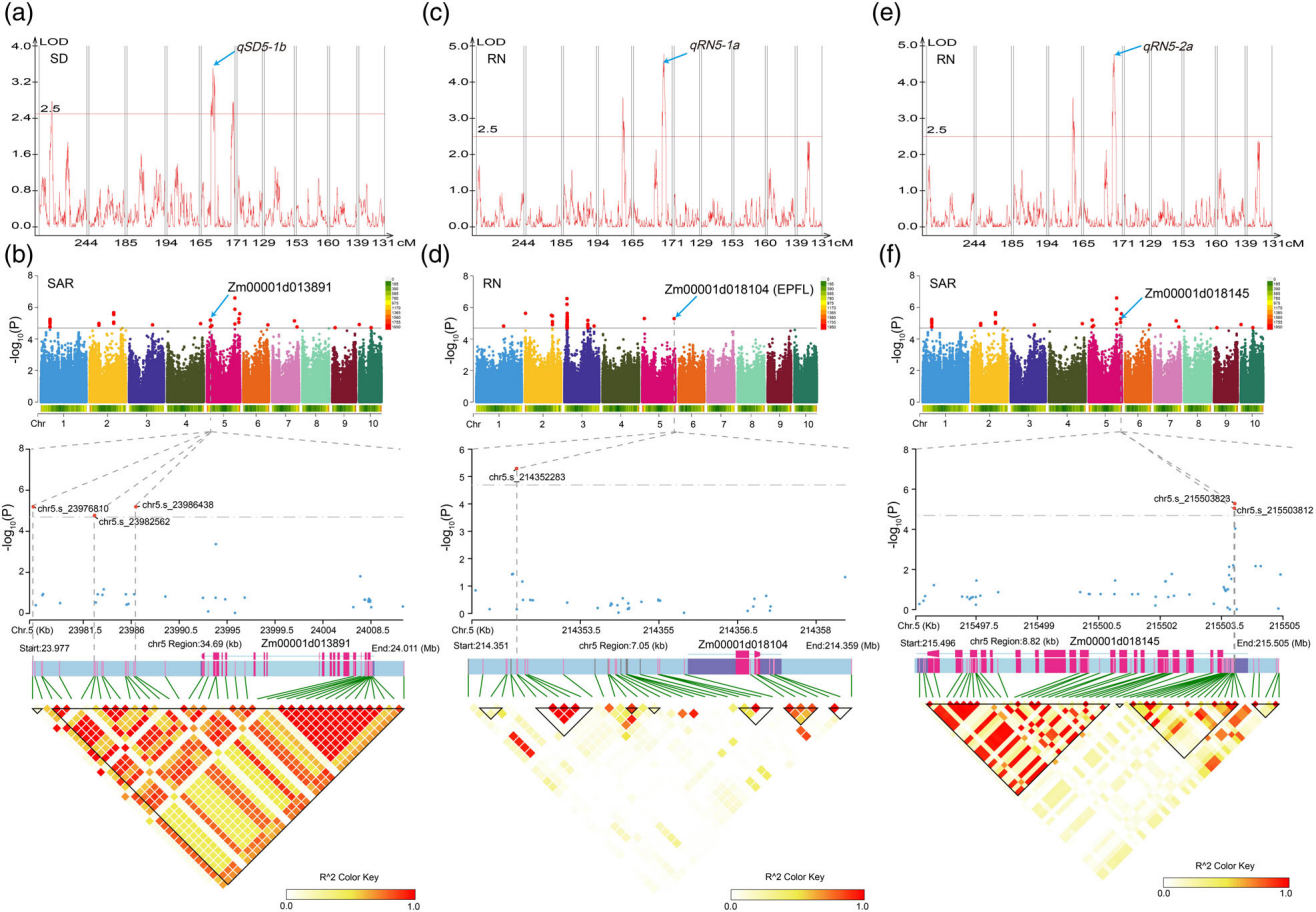
Co‐localized quantitative trait loci (QTL) identified by linkage and association mapping. (a, c, e) Linkage mapping results for stomatal traits, showing stomatal density (SD) in the BYK (a) population, and stomatal row number (RN) in the BYD population (c, e). QTL intervals detected in multiple populations are indicated by blue arrows. (b, d, f) Integrated association mapping views for the corresponding co‐localized QTL intervals. For each locus, the top panel shows the Manhattan plot for the respective trait (SD in b, RN in d and f); the middle panel displays the single‐nucleotide polymorphism (SNP) density across the interval, and the bottom panel shows the pairwise linkage disequilibrium (LD) pattern. The lead genome‐wide association study (GWAS) SNP is highlighted (red dot), and its genomic position relative to a candidate gene is indicated. The gray dashed line denotes the genome‐wide significance threshold (*P* = 2.04E‐05). Red dots in the linkage mapping panels (a, c, e) indicate lead GWAS SNPs that fall within the corresponding QTL intervals.

### Prioritization of candidate genes within QTL intervals

To extract biological insight from the QTL identified for stomatal traits, we annotated candidate genes within linkage‐ and association‐based intervals and applied a prioritization strategy that integrated homology to known stomatal regulators, functional annotation, and cell‐type‐specific expression patterns (Sun et al., 2022; Zhang et al., 2023). This approach substantially reduced the candidate space and enabled focused analysis of genes most likely to contribute to natural variation in stomatal development. In total, 40 and 67 core candidate genes were retained for association and linkage populations, respectively, with their gene ID shown in bold in Tables S6 and S7, respectively. We therefore concentrated on QTL that were repeatedly detected across mapping populations, exhibited potential pleiotropic effects on multiple traits, or were independently supported by both linkage mapping and GWAS (Tables S6 and S7).

This prioritization yielded three main functional categories. One is the conserved stomatal regulators. Several high‐confidence QTL intervals across multiple populations contained maize orthologs of well‐established stomatal development genes. Within a potential pleiotropic interval on chromosome 4 affecting RN and size‐related traits (Figure 2; Figures S2 and S3), we identified a bHLH transcription factor homologous to Arabidopsis SPEECHLESS (MacAlister et al., 2007; Pillitteri et al., 2007; Renard et al., 2020). QTL intervals associated with SD and SAR included *EPIDERMAL PATTERNING FACTOR‐LIKE* (*EPFL*) gene (Figure 3c–f; Table S7) (Shpak et al., 2005; Takata et al., 2013; Xiong et al., 2022), and the interval on chromosome 2 affecting RN and size‐related traits contained GRAS family members related to SCARECROW (Table S7) (Hughes et al., 2023; Shaar‐Moshe & Brady, 2023). Cell wall biosynthesis and modification is another category. Multiple robust QTL intervals affecting SD, size, and RN contained genes encoding cellulose synthase and xylan biosynthesis components, including CESA6 and IRX15A, as well as enzymes implicated in polysaccharide modification (Table S7) (Brown et al., 2011; Huang et al., 2020; Jensen et al., 2011; Zhang et al., 2023). Signaling and metabolic genes are also emphasized. A smaller subset included genes encoding sucrose transporters expressed in guard cells and ion channel‐related proteins implicated in calcium signaling (Kudla et al., 2010; Lawson & Matthews, 2020; Murata et al., 2015), detected within QTL intervals affecting SD and SAR (Table S7). Based on these prioritized candidates and their contrasting predicted functions, we selected two genes, *ZmbHLH140* (an annotated bHLH transcription factor) and *ZmCESA6* (a cellulose synthase), for functional validation.

### Functional validation of candidate genes regulating stomatal traits

*ZmbHLH140* was prioritized from a QTL interval associated with SD (Figure 2; Figures S5 and S6; Table S6). Phylogenetic analysis showed that ZmbHLH140 falls outside the clade of known stomatal bHLH regulators (SPCH, MUTE, FAMA, ICE1/SCRM), ROX, and other Arabidopsis bHLH members, but is most closely related to AtAPTX (involved in DNA repair) (Figure S8). To assess its function, an EMS‐induced allele carrying a premature termination mutation in *ZmbHLH140* was identified (Figure 4a). Homozygous *Zmbhlh140* mutants exhibited significantly reduced SD, RN, and stomatal index (SI) compared with wild‐type siblings, coupled with increased SL but unchanged SW and SA (Figure 4b–h). These results position *ZmbHLH140* as a positive regulator of SD and RN, required for normal maize stomatal development. To assess the relevance of *ZmbHLH140* to natural variation in maize, we examined allelic diversity at this locus in the 262 inbred line association panel. Candidate gene association analysis identified two non‐synonymous SNPs (SNP2347 and SNP3355) significantly associated with SD (Figure 4i). Haplotype analysis revealed distinct allelic classes with contrasting effects on SD, with accessions carrying the favorable haplotype exhibiting increased SD relative to other haplotypes (Figure 4j). Consistent with a role in maize improvement, analysis of selection using HapMap3 data revealed a marked reduction in polymorphism at the *ZmbHLH140* locus in maize relative to teosinte (*P* = 2.64E‐11, locus‐specific test comparing maize and teosinte alleles; see Methods), a negative Tajima's *D*, and a moderate elevation in both Fst and XP‐CLR (Figure 4k–n). However, neither Fst nor XP‐CLR exceeds the empirical genome‐wide outlier thresholds (95 and 99% percentiles). The convergence of these multiple statistics, though each individual signal is moderate, is consistent with positive selection during domestication, particularly when considered alongside the functional evidence described above. Together, these results demonstrate that *ZmbHLH140* acts as a positive regulator of SD and RN and that natural variation at this locus has contributed to phenotypic diversification during maize evolution, although definitive causal inference will require additional functional validation.

**Figure 4:**
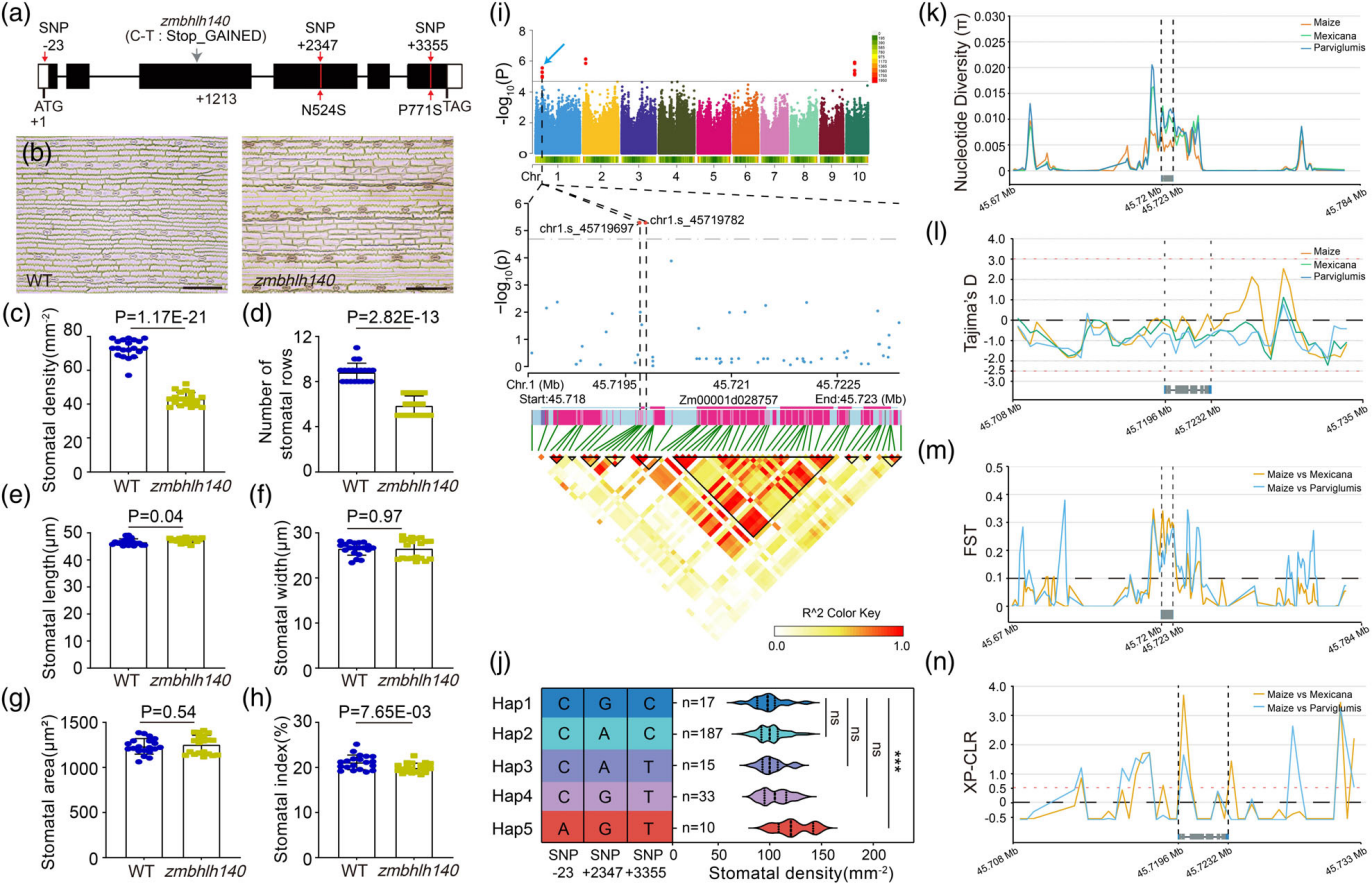
Loss of function of *ZmbHLH140* reduces stomatal density in maize. (a) Gene structure of *ZmbHLH140* showing the EMS‐induced premature termination mutation (arrow). Lines, introns; black boxes, coding sequences (CDS); white boxes, untranslated regions (UTRs). Three naturally occurring non‐synonymous variants in the coding region are highlighted with red arrows. (b) Representative micrographs of the abaxial leaf epidermis from three‐leaf‐stage WT and *zmbhlh140* mutant plants. Scale bar = 200 μm. (c, d) Quantitative comparisons of stomatal density (c) and stomatal row number (d) between WT and *zmbhlh140* mutants. (e–g) Quantitative comparisons of stomatal size between WT and *zmbhlh140* mutants. Each data point denotes the average stomatal dimension of a single leaf micrograph. (h) Quantitative comparisons of stomatal index between WT and *zmbhlh140* mutants. For (c–h), data are presented as mean ± SD; *n* = 20 micrographs from four plants (five images per plant). Statistical significance was determined by Student's *t*‐test; *P*‐values are shown in scientific notation, and *P* < 0.05 was considered significant. (i) Regional association mapping plot focused on the *ZmbHLH140* genomic locus on chromosome 1. The lead single‐nucleotide polymorphism (SNP) is marked in red. The 4.5‐kb region flanking the most significant SNP is shown. (j) Major haplotypes of *ZmbHLH140* identified in the association panel. The number of accessions (*n*) per haplotype is shown. Violin plots display the distribution of stomatal density (SD) for each haplotype. Significant differences between haplotypes were assessed by a two‐sided *t*‐test (*** indicates *P* < 0.001). (k–n) Population selection analysis of *ZmbHLH140*. Sliding‐window profiles of nucleotide diversity (*π*), FST, Tajima's *D*, and XP‐CLR across the *ZmbHLH140* region in 81 Mexican teosinte accessions, 71 small‐glume teosinte accessions, and 507 maize inbred accessions.

To assess how natural allelic variation in cell wall biosynthesis influences stomatal traits, we next characterized *ZmCESA6*, which encodes a cellulose synthase involved in primary cell wall formation and was associated with variation in SD and RN. An EMS‐induced allele revealed a G‐to‐A mutation in exon 3, which was predicted to produce a truncated protein (Figure 5a). In contrast to *bhlh140*, homozygous *zmcesa6* mutants exhibited a significant increase in both SD and RN but a significant decrease in SL, SW, SA, and unchanged SI relative to wild‐type siblings (Figure 5b–h). This opposing phenotype suggests that *ZmCESA6* plays a distinct role in modulating stomatal patterning, possibly through its involvement in cellulose biosynthesis and cell wall assembly. Additionally, unlike *ZmbHLH140*, no clear selection signature at *ZmCESA6* locus during domestication was observed, despite a marked reduction in polymorphism in multiple selection statistics (Figure S9). This pattern may reflect purifying selection or demographic effects, but its interpretation remains uncertain without additional population genomic analyses. Together, these results support a model in which SD is shaped by the integration of regulatory networks controlling cell identity and cell wall–mediated growth constraints.

**Figure 5:**
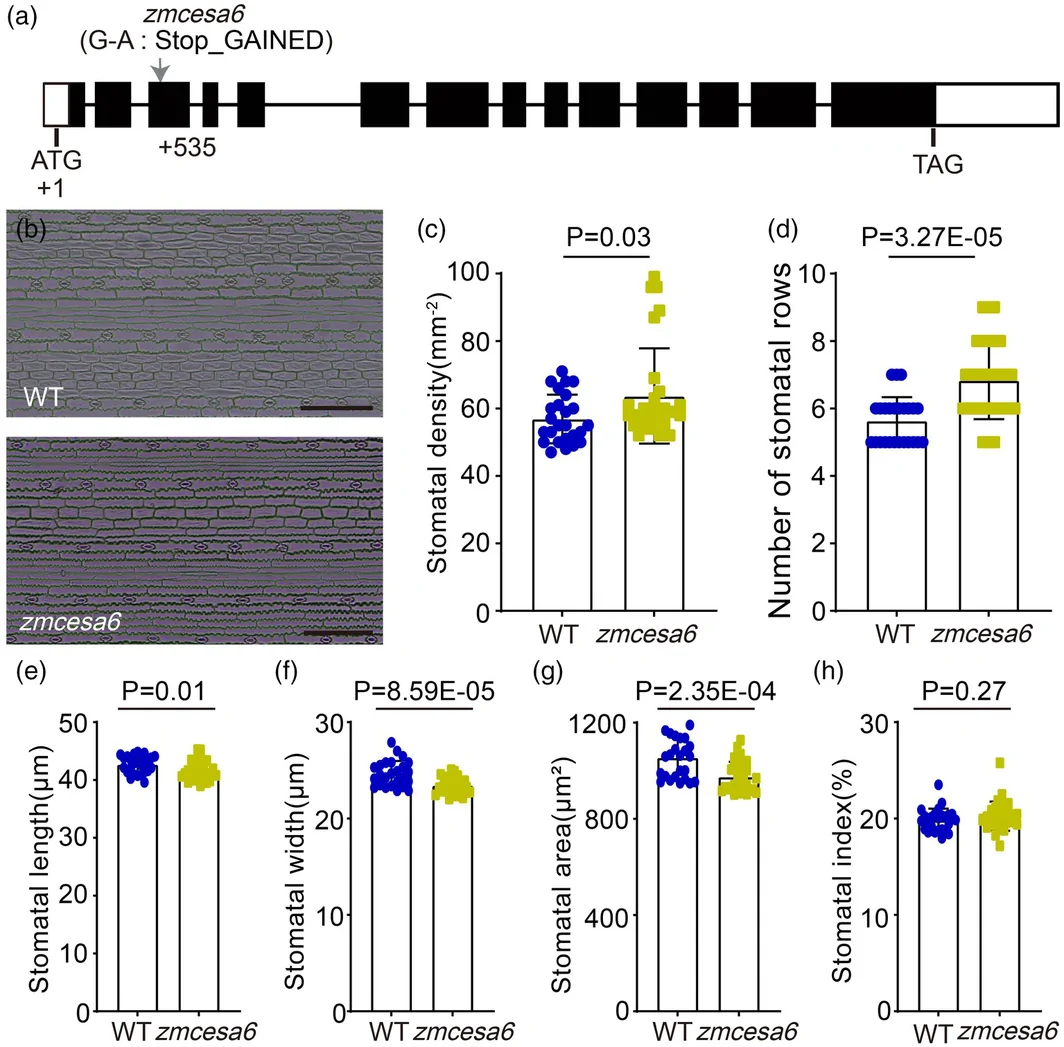
Loss of function of *ZmCESA6* increases stomatal density in maize. (a) Gene structure of *ZmCESA6* showing the site of the EMS‐induced mutation (arrow). Lines, introns; black boxes, coding sequence (CDS); white boxes, untranslated regions (UTRs). (b) Representative micrographs of the abaxial leaf epidermis from three‐leaf‐stage wild‐type and *zmcesa6* mutant plants. Scale bar = 100 μm. (c, d) Quantitative comparisons of stomatal density (c) and stomatal row number (d) between WT and *zmcesa6* mutants. (e–g) Quantitative comparisons of stomatal size between WT and *zmcesa6* mutants. (h) Quantitative comparisons of stomatal index between WT and *zmcesa6* mutants. For (c–h), data are presented as mean ± SD; *n* = 25 micrographs from five wild‐type plants and *n* = 30 micrographs from six mutant plants (five images per plant). Statistical significance was determined by Student's *t*‐test; *P*‐values are shown in scientific notation, and *P* < 0.05 was considered significant.

## DISCUSSION

Understanding how stomatal traits are genetically controlled remains a central challenge in plant biology. Although extensive natural variation in SD, SS, and arrangement has been documented across plant species, linking this variation to specific genetic mechanisms—particularly in crops—has proven difficult. A long‐standing obstacle has been disentangling whether SD and SS are independently tunable traits or whether they are constrained by shared developmental and genetic programs. Resolving this question is essential for interpreting stomatal evolution and for understanding the developmental constraints that shape stomatal diversity. Here we addressed this question by combining linkage and association mapping in maize, complemented by transcriptomic prioritization and mutant analysis.

A defining feature of stomatal biology is the inverse relationship between SD and SS, which represents a fundamental ecological trade‐off (Caine et al., 2023; Franks & Beerling, 2009). While this trade‐off has been repeatedly observed at the phenotypic level, direct genetic evidence explaining its origin has been limited. Our integrated mapping analyses provide such evidence by demonstrating that the SD‐SS trade‐off in maize is genetically correlated (Figure 1; Figure S1). We identified potential multiple pleiotropic QTL that coordinately influence both SD and size‐related traits (Figure 2; Table S5), indicating that natural variation at single loci may shift stomatal developmental toward strategies favoring either numerous small stomata or fewer larger ones. However, without fine‐mapping or NIL analyses, QTL co‐localization alone cannot distinguish true pleiotropy from tight linkage. Thus, linked but functionally independent genes remain a plausible alternative. Moreover, all phenotyping was performed in a single field environment, which raises the possibility that some identified loci may be environment‐specific. Multi‐environment trials, physiological assays, and higher resolution mapping will therefore be essential to confirm the robustness of these QTL and to disentangle environmental effects on stomatal development. Additionally, although we adopted a conservative overlap criterion (GWAS SNPs within 2‐LOD QTL intervals), the absence of a formal permutation test for the significance of interval overlap, combined with the limited detection power of our 262‐line panel given maize's rapid linkage disequilibrium (LD) decay, means that the six shared intervals are only suggestive. Positional comparison with the independent mapping study of Xie et al. (2021) further supports this caution, 5 of the 33 robust QTL intervals and 16 of the 51 association loci overlapped intervals reported in that study. Given the differences in germplasm and environment between the two studies and the limited cross‐year replication rates reported previously, an overlap of approximately 11.6% and approximately 31.4% with a single study is moderate and consistent with expectation. This supports the general positional conservation of stomatal/drought‐related regions rather than exact per‐locus co‐localization, and these regions may be driven by different genes or alleles in different genetic backgrounds. In contrast to density‐centric effects, our identified loci may also influence gas exchange indirectly or through interactions with other traits (e.g., stomatal kinetics and anatomy), as suggested by previous studies (Crawford et al., 2025; Xie et al., 2021). This underscores the need for integrative approaches that bridge genetic variation to dynamic stomatal performance. Notably, stomatal arrangement ratio (SAR) emerged as a genetically distinct trait, showing low correlation with SD and SS across all populations but yielding the second‐most GWAS hits, implying independent genetic control. Biologically, SAR may capture the developmental plasticity of stomatal initiation along the leaf axis, offering an underexplored angle on stomatal patterning.

The identification of conserved stomatal regulators within robust QTL intervals provides biological support for the genetic architecture we uncovered. Orthologs of core Arabidopsis genes involved in stomatal lineage initiation and patterning, including *SPEECHLESS*/*SPCH*, *EPIDERMAL PATTERNING FACTOR‐LIKE* (*EPFL*) peptides, and *SCARECROW* (*SCR*)‐related GRAS transcription factors, were repeatedly detected across mapping populations (Figures 2 and 3; Tables S6 and S7). These regulators are known to govern stomatal lineage initiation (MacAlister et al., 2007; Pillitteri et al., 2007) and stomatal formation (Li et al., 2023; Takata et al., 2013) in both eudicots and grasses. Their presence within maize QTL intervals indicates that natural allelic variation in deeply conserved developmental pathways contributes substantially to stomatal trait diversity. This conservation supports the genetic architecture uncovered here and suggests that evolutionary divergence in stomatal traits often operates through modulation of core regulatory modules rather than through entirely novel mechanisms.

Beyond transcriptional and signaling networks, our analysis highlights cell wall biosynthesis and modification as critical, yet underappreciated, determinants of stomatal morphology in grasses. Several high‐confidence QTL intervals contained genes involved in cellulose and hemicellulose biosynthesis, including *ZmCESA6* and *IRX15A*, as well as additional enzymes implicated in polysaccharide modification, all of which were corroborated by both linkage and association analysis in this study and previous reports (Figure 2; Sun et al., 2022; Zhang et al., 2023). IRX15A, in particular, has been shown to participate in xylan biosynthesis and secondary cell wall formation, processes that influence cell wall architecture and mechanical properties. The importance of these pathways is particularly relevant in grasses, where dumbbell‐shaped guard cells and their precise coordination with subsidiary cells impose unique mechanical constraints. Proper wall deposition, rigidity, and anisotropic expansion are therefore essential not only for guard cell function but also for defining SS and spacing. Allelic variation in genes regulating primary and secondary cell wall assembly is thus likely to modulate these mechanical properties, providing a mechanistic basis for the pleiotropic effects observed across multiple stomatal traits.

In addition to these core developmental and mechanical pathways, our prioritization also identified a smaller but noteworthy category of candidate genes linking stomatal patterning to broader physiological processes. Specifically, we detected genes encoding sucrose transporters expressed in guard cells and ion channel‐related proteins implicated in calcium signaling within QTL intervals affecting SD and SAR (Table S7). Although their direct roles in stomatal development remain to be experimentally defined, their presence raises the possibility that the developmental programs governing stomatal patterning may be coordinated with guard cell metabolic and signaling capacity (Kudla et al., 2010; Lawson & Matthews, 2020; Murata et al., 2015). Such integration could provide a mechanism by which stomatal initiation and spacing are tuned to physiological demand during epidermal differentiation, a hypothesis that warrants future investigation.

Functional validation of two contrasting candidate genes, *ZmbHLH140* and *ZmCESA6*, further illustrates how distinct genetic pathways converge on stomatal patterning (Figures 4 and 5), although independent alleles would provide much stronger genetic evidence. Loss of function of *ZmbHLH140* reduced SD, RN, and SI, while *ZmCESA6* mutation increased SD and RN but not SI. Size phenotyping further reveals a gene‐specific trade‐off; *ZmbHLH140* loss decreased density but increased length only (width and area unchanged), a partial trade‐off, whereas *zmcesa6* exhibited increased density with reductions in length, width, and area, fully consistent with the canonical trade‐off. Thus, *ZmCESA6* exemplifies complete pleiotropy across size parameters, while *ZmbHLH140* exerts partial or indirect effects, indicating that the mechanistic link between number and size differs among pathways. However, these conclusions derive from mutant analyses at a single developmental stage (three‐leaf stage), and the extent to which they reflect the QTL‐level genetic correlations observed in natural populations, versus potentially distinct allelic effects at the same loci, remains unsolved. Regarding selection at *ZmbHLH140*, the convergent signals (reduced diversity, negative Tajima's *D*, and moderate Fst/XP‐CLR) are suggestive of positive selection, but none of the individual outlier statistics reach genome‐wide thresholds. We therefore interpret this evidence as suggestive rather than definitive, and further functional validation in diverse genetic backgrounds will be needed to establish a causal role in domestication‐related traits. Given that stomatal traits differ between juvenile and mature leaves, future validation in mature leaves is necessary. Moreover, the physiological impacts of mutations in these regulatory genes, such as alterations in gas exchange, stomatal conductance, or water‐loss dynamics, should be evaluated under field conditions to inform practical breeding or management strategies. Additionally, SI quantification was not feasible for QTL populations because the original images focused solely on stomata, leaving pavement cells indistinct; consequently, our SI measurements were obtained only from mutant lines, and the correlation between SI and QTL variation remains unknown. Finally, direct phenotypic and genetic correlation analyses between stomatal traits and drought resistance or yield are not available within our current experimental framework. Nevertheless, we have systematically compared our stomatal QTL with previously reported drought‐tolerance QTL (Almeida et al., 2013, 2014; Li et al., 2016; Semagn et al., 2013; Wu et al., 2021), providing indirect genetic evidence; however, multi‐trait phenotyping is required to establish direct genetic correlations.

In summary, this study establishes a comprehensive genetic framework for stomatal trait variation in maize by integrating linkage mapping, association analysis, transcriptomic prioritization, and functional validation. Our findings suggest that the classic trade‐off between SD and SS may be influenced by pleiotropic genetic loci, conserved developmental regulators, and cell wall–mediated mechanical control, although the extent of pleiotropy is gene‐dependent, with complete trade‐offs in some cases (*ZmCESA6*) and partial effects in others (*ZmbHLH140*). By revealing how these processes interact to shape stomatal patterning, this work advances our understanding of stomatal development in grasses and provides a foundation for future functional validation of the genetic regulators identified here.

## METHODS

### Plant materials and field trials

A diverse panel of 262 maize inbred lines (Yang et al., 2010), representing germplasm from China, the International Maize and Wheat Improvement Center (CIMMYT), and the United States, was used for GWAS. This panel encompassed materials adapted to tropical, subtropical, and temperate climates, including key inbred lines widely used in contemporary Chinese maize breeding programs (Table S8). The association panel was classified into four subgroups: mixed (79 lines), non‐stiff stalk (NSS; 105 lines), stiff stalk (SS; 26 lines), and tropical–subtropical (TST; 52 lines).

Three RIL populations, BYK (K22 × By815, 163 lines), BYD (DE3 × By815, 142 lines), and SZ (Z58 × SK, 105 lines), previously described by Pan et al. (2016), were employed for linkage mapping. All materials were grown during the 2023 growing season in Sanya, Hainan, China (N18.25°, E109.51°), using a randomized complete block design. Each plot consisted of a 4‐m row with 25 cm spacing between plants and 67 cm between rows, adhering to standard agronomic practices.

### Stomatal phenotyping

To avoid leaf‐age‐induced variation, the middle section of ear leaves at silking stage was uniformly collected for stomatal phenotypic measurement. Stomatal traits were quantified using a nail polish impression method as previously described (Ceulemans et al., 1995; Miyazawa et al., 2006; Reeves et al., 2018; Xie et al., 2022; Zhao et al., 2016, 2022). Briefly, the abaxial epidermis from the midsection of fully expanded leaves, equidistant from the midrib and leaf margin, was gently cleaned and coated with clear nail varnish. For each genotype, five individual plants was sampled, with eight impressions collected per leaf. After drying, the varnish films were peeled, mounted on glass slides, and covered with coverslips (Zhang et al., 2022). Stomatal impressions were imaged using an Olympus BX43 microscope. SL, SW, SA, the number of stomatal rows (RN), and stomatal arrangement ratio (SAR), were quantified from digital images using a custom image‐analysis interface. SD was calculated as the number of stomata per unit leaf area (μm^2^) (Radoglou & Jarvis, 1990). SL was defined as the maximum dimension parallel to the guard cell pair, and SW was measured as the maximum width perpendicular to the long axis of the stomatal apparatus (Zhou et al., 2023). SAR was calculated as the ratio of the total stomatal number (*N*_extra_ + *N*_basic_) to the basic stomatal number (*N*_basic_) in a single stomatal row. For each genotype, 5 plants were measured with 8 observations per plant, giving 40 observations in total. Raw phenotypic data were curated via Microsoft Office 2021. Outliers were removed based on trait‐specific thresholds, for SD, RN, and SAR, count‐based traits with skewed distributions and variance scaling with the mean, a dispersion‐based exclusion of ±3 standard deviations (SD) was used; for SL, SW, and SA, that vary continuously across developmental stages, a fixed proportional window of ±30% of the trait mean was used. For each trait, measurements from individual plants were first averaged across all impressions after outlier removal; the resulting per‐plant values were then averaged to obtain the genotype mean used for QTL mapping.

### Statistical analysis and QTL mapping

Phenotypic data were compiled in Microsoft Excel and analyzed using SPSS Statistics (version 22; IBM Corp., Chicago, IL, USA). Data normality and homogeneity of variance were assessed using the Kolmogorov–Smirnov test and Levene's test, respectively.

Ultra‐high‐density genetic maps of the three RIL populations were obtained from Pan's study (Pan et al., 2016), which were converted to physical distance in B73‐V4 version using the Gramene database genome coordinate conversion tools (https://ensembl.gramene.org/Oryza_sativa/Tools/AssemblyConverter?db=core). QTL mapping was performed using composite interval mapping (Model 6) implemented in Windows QTL Cartographer version 2.5 (Zeng, 1994), with a walk speed of 0.5 cM and a window size of 10‐cM. QTL detection was initiated using a nominal LOD threshold of 2.5, with individual QTL intervals delimited by a 2.0 LOD drop from the peak marker. To establish robust, environment‐ and trait‐specific significance levels, we performed 1000 permutation tests (*P* = 0.05), which yielded LOD thresholds ranging from 3.1 to 3.6 across different conditions (Table S5). Additionally, QTL with LOD scores falling between the nominal threshold and the permutation‐derived stringent thresholds were conditionally retained, provided they had also been independently documented in at least three published studies focusing on stomatal patterning traits (Xie et al., 2021) or drought‐stress responses (Almeida et al., 2013, 2014; Li et al., 2016; Semagn et al., 2013; Wu et al., 2021). To establish a chance baseline for the conditional retention criterion, we performed permutation analyses with regioneR package using the union of all six published studies as the reference set. The physical positions of all QTL were anchored to the B73 reference genome (version 4). For interval merging, adjacent QTL sharing boundary markers within their peak intervals were consolidated into a single locus if the LOD valley between adjacent peaks remained above the significance threshold. Additionally, two QTL were deemed co‐localized when their physical overlap exceeded 10% of the length of the shorter interval. QTL was designed according to the following format, q + trait abbreviation + chromosome number + numberic identifier + population code, where the population code ‘a’ refers to the BYD population, ‘b’ to the BYK population, and ‘c’ to the SZ population. Candidate genes within these intervals were identified based on the B73 RefGen_v4 gene set from MaizeGDB (http://www.maizegdb.org) (Jiao et al., 2017) and functionally annotated using InterProScan (http://www.ebi.ac.uk/interpro/scan.html; Jones et al., 2014).

### Genome‐wide association study

Genotype data for the association panel consisted of 1.25 Mb high‐quality SNPs with a minor allele frequency (MAF) ≥0.05 (Liu et al., 2017). GWAS was conducted using a mixed linear model (MLM) in TASSEL 5.0 (Bradbury et al., 2007), which incorporated kinship (*K* matrix) and population structure (*Q*) to control for spurious associations. The genome‐wide significance threshold was set at *P* = 2.04E‐05 based on the effective number of independent SNPs. Manhattan and quantile‐quantile (Q‐Q) plots with genomic inflation factor (*λ*) were generated using the ‘CMplot’ package in R (Yin et al., 2021). Significant association loci were defined based on the LD decay distance (approximately 30 kb, *r* ^*2*^ = 0.1), with non‐redundant association loci designated as 60‐kb regions (30 kb upstream and downstream) surrounding each significant SNP. Co‐localization between the two populations was defined when the physical coordinates of GWAS‐derived significant SNP loci were fully contained within the confidence intervals of QTLs mapped via linkage analysis.

### Population selection analysis

The domestication and selection characteristics of *ZmbHLH140* and *ZmCESA6* were analyzed using three population panels: 81 Mexican teosinte accessions, 71 small‐glume teosinte accessions, and 507 maize inbred lines. Selection scans were performed using a sliding‐window approach with a window size of 1000 bp and a step size of 500 bp. Nucleotide diversity (*π*) and Fst were calculated using VCFtools v0.1.16 (Danecek et al., 2011), and XP‐CLR was computed following Chen et al. (2010). Tajima's *D* was calculated using the PopGenome package in R. The genome‐wide empirical thresholds for Fst and XP‐CLR were determined as the 95 and 99% percentiles of the respective genome‐wide distributions. The *P*‐value for reduced polymorphism derives from a locus‐specific comparison of maize versus teosinte alleles rather than from a genome‐wide outlier test against the empirical null distribution.

### Candidate gene annotation and functional validation

Candidate genes within QTL and GWAS intervals were retrieved from the MaizeGDB (https://www.maizegdb.org/gbrowse) and Uniprot (https://www.uniprot.org/) databases based on the B73 RefGen_v4 reference. Priority was given to genes fit to the following criteria: (1) expressed in maize leaf tissues (FPKM >1 in maize leaf tissues) (Stelpflug et al., 2016); or (2) exhibiting high expression levels in young leaves (The expression ratio between immature leaves and roots is greater than 0.5); and (3) annotated as stomatal‐related differentially expressed genes from published maize stomata single‐nucleus transcriptome (adjusted *P* < 0.05) (Sun et al., 2022); or (4) previously reported to be associated with stomatal traits in other species used only as auxiliary evidence.

For functional validation, EMS‐induced mutants of *ZmbHLH140* (EMS4‐008220) and *ZmCESA6* (EMS5‐056202) in the B73 background were obtained from the Maize EMS‐induced Mutant Database (http://www.elabcaas.cn/memd/). Homozygous mutant and wild‐type sibling plants were identified by locus‐specific genotyping. To reduce the background mutation load associated with the EMS mutagenesis, heterozygous carriers were backcrossed twice to the B73 wild‐type parent, followed by two generations of self‐pollination. From the resulting progeny, homozygous mutant and homozygous wild‐type siblings derived from the same selfed heterozygous family were selected for phenotypic evaluation, with three independent sibling families assessed. This sibling‐based design effectively minimizes confounding effects arising from residual background mutations. Stomatal phenotyping was performed on the abaxial epidermis at a position three‐quarters of the distance from the tip of the third leaf at the V3 stage (10 days after germination), using the nail polish impression method described above. To separate changes in SD from changes in epidermal cell size, SI (stomata/total epidermal cells) was also evaluated. The primers used for genotyping are listed in Table S9.

## AUTHOR CONTRIBUTIONS

ZF and JT planned and designed the research. ZF, YQ, MQ, ZP, LS, ZY, XM, CW, TW, YH, and HW performed the experiments, conducted fieldwork, and generated the data. ZF and QZ analyzed the data, wrote and revised the manuscript. YQ, MQ, and CW contributed equally.

## CONFLICT OF INTEREST

The authors declare no competing interests.

## Supporting information


**Figure S1.** Correlation analysis of stomatal traits in additional RIL populations.

**Figure S2.** Linkage mapping reveals QTL controlling stomatal traits.

**Figure S3.** Detailed QTL maps from linkage analysis.

**Figure S4.** Permutation test of overlaps between linkage‐mapped QTL intervals and published stomata‐ and drought‐related QTL.

**Figure S5.** Genome‐wide association study identifies QTL associated with stomatal traits.

**Figure S6.** Genome‐wide association study results for all stomatal traits.

**Figure S7.** Permutation test of overlaps between GWAS‐derived QTL intervals and published stomata‐ and drought‐related QTL.

**Figure S8.** Phylogenetic analysis of ZmbHLH140.

**Figure S9.** Selection analysis of *ZmCESA6*.

**Table S1.** Stomatal phenotypic data of linkage population.
**Table S2.** Stomatal phenotypic data of association population.
**Table S3.** Variation in phenotypic traits across three linkage populations.
**Table S4.** Variation in phenotypic traits within the association mapping population.
**Table S5.** Summary of QTL identified in three recombinant inbred line (RIL) populations.
**Table S6.** QTLs identified in the association population and their co‐localization with previously reported QTLs.
**Table S7.** Candidate genes located within QTL identified across three RIL populations.
**Table S8.** List of inbred lines used for association mapping in this study.
**Table S9.** Primers used in this study.

## Data Availability

The data that support the findings of this study are available in the Supporting Information or on request from the corresponding author.
